# The Prognostic and Immunological Value of Guanylate-Binding Proteins in Lower-Grade Glioma: Potential Markers or Not?

**DOI:** 10.3389/fgene.2021.651348

**Published:** 2021-10-25

**Authors:** Zhuang Liu, Jifeng Sun, Ting Gong, Huixin Tang, Yanna Shen, Chang Liu

**Affiliations:** ^1^ Department of Biochemistry and Molecular Biology, Key Laboratory of Breast Cancer Prevention and Therapy, Ministry of Education, Tianjin Medical University Cancer Institute and Hospital, National Clinical Research Center for Cancer, Key Laboratory of Cancer Prevention and Therapy, Tianjin’s Clinical Research Center for Cancer, Tianjin, China; ^2^ Department of Radiation Oncology, Tianjin Cancer Hospital Airport Hospital, Tianjin, China; ^3^ Department of Oncology, Tianjin Medical University General Hospital, Tianjin, China; ^4^ School of Medical Laboratory, Tianjin Medical University, Tianjin, China

**Keywords:** guanylate-binding proteins, lower-grade glioma, prognosis, immune infiltration, pathway analysis

## Abstract

Seven guanylate-binding proteins (GBPs, GBP1–7), identified as a subfamily of interferon-γ-induced guanosine triphosphate hydrolases (GTPases), has been reported to be closely associated with tumor progression, metastasis, and prognosis of cancer patients in recent years. However, the expression patterns, prognostic value, immune infiltration relevance, and biological functions of GBPs in lower-grade glioma (LGG) remain elusive. In this study, by analysis and verification through multiple public data platforms, we found that GBP1, 2, 3, 4 were significantly upregulated in LGG tissues vs normal brain tissue. Analysis based on the Cox proportional hazard ratio and Kaplan–Meier plots demonstrated that the high expressions of GBP 1, 2, 3, 4 were significantly correlated with the poor prognosis of LGG patients. Correlation analysis of clinical parameters of LGG patients indicated that the expressions of GBP 1, 2, 3, 4 were significantly associated with the histological subtype and tumor histological grade of LGG. Furthermore, the correlation analysis of immune infiltration showed that the expressions of GBP1, 2, 3, 4 were significantly and positively correlated with the level of tumor immune-infiltrating cells. In particular, GBP1, 2, 3, 4 expressions were strongly correlated with the infiltration levels of monocyte, TAM, and M1/M2 macrophage, revealing their potential to regulate the polarity of macrophages. Finally, we used the GSEA method to explore the signaling pathways potentially regulated by GBP1, 2, 3, 4 and found that they were all closely associated with immune-related signaling pathways. Collectively, these findings suggested that GBP1, 2, 3, 4 were potent biomarkers to determine the prognosis and immune cell infiltration of LGG patients.

## Introduction

Glioma is derived from astrocytes and/or oligodendrocytes and is one of the most common primary central nervous system tumors (Jang and Kim, 2018). Lower-grade glioma (LGG), the crucial pathological type of glioma, comprises grade II and grade III gliomas defined by the World Health Organization (WHO), mainly including anaplastic astrocytomas, oligodendrogliomas, and oligoastrocytomas (Brat et al., 2015). LGG has the characteristics of diffuse infiltration, metastasis, and easy progression to higher-grade gliomas, which seriously affects human survival, especially young adults who enjoy an active life (Mazurowski et al., 2017; Liu et al., 2019). In recent years, comprehensive treatments such as postoperative chemotherapy, radiotherapy, and immunotherapy have made great progress, but the survival rate of LGG patients is still unsatisfactory and unpredictable. Thus, the identification of novel prognostic biomarkers or molecular targets is imperative for a highly accurate prediction of survival or guidance for individualized treatment of LGG patients.

Guanylate-binding protein (GBP) is classified as a unique subfamily of interferon-γ-induced guanosine triphosphate hydrolases (GTPases), which can hydrolyze guanosine triphosphate (GTP) to both guanosine diphosphate (GDP) and guanosine monophosphate (GMP) (Ghosh et al., 2006). In humans, seven GBP proteins (GBP1–7) with molecular weights in the range of 67–73 kDa have been well identified (Tripal et al., 2007). Studies have shown that GBPs, such as GBP1 and GBP2, are closely related to host defense against pathogens, and have antiviral and antibacterial activities in the process of host anti-infection and anti-inflammatory defense (Vestal and Jeyaratnam, 2011; Honkala et al., 2019). However, the roles of GBPs in cancer are diverse and complicated. GBP1 upregulation is reported to be associated with decreased disease progression and better overall survival in patients with breast and colorectal cancer (Naschberger et al., 2008; Lipnik et al., 2010), while it is connected with increased disease progression, metastasis, and treatment resistance in ovarian cancer and glioblastoma (Duan et al., 2006; De Donato et al., 2012; Ji et al., 2019). GBP2 can enhance the invasion of glioblastoma (Yu et al., 2020), but inhibit the invasion ability of breast cancer cells (Zhang et al., 2017). Thus, the functions of different GBPs in multiple cancers need to be further clarified.

The immune microenvironment has been determined to play a vital role in tumor biology (Zhang et al., 2020). Tumor-infiltrating immune cells, including T and B lymphocytes, macrophages, neutrophils, dendritic cells, etc., are very important elements of the tumor microenvironment, which directly or indirectly regulate the growth and development of tumor cells and further affect the prognosis of many cancer patients including LGG (Domingues et al., 2016; Zhang and Zhang, 2020). Recently, many promising preclinical and clinical immunotherapies have been implemented in malignant glioma, including immune checkpoint inhibitors, active or passive immunotherapy, etc. (Simonelli et al., 2018; Vismara et al., 2019), indicating that the immune components in the tumor microenvironment are of great value as prognostic biomarkers or therapeutic targets in glioma. Therefore, further exploration of immune infiltration regulation in the tumor microenvironment may support the treatment of cancers.

However, there are relatively a few studies on GBPs in LGG, and the prognostic value, the regulation of immune infiltration, and biological functions of GBPs in LGG need to be further clarified. In this study, we used public databases and online platforms and conducted a comprehensive and detailed analysis of the expression patterns, prognostic value, immune infiltration regulation, and biological functions of GBPs in LGG.

## Methods

### Data Collection

RNAseq data and corresponding clinical data of 509 LGG tissue samples were downloaded from TCGA (The Cancer Genome Atlas, https://portal.gdc.cancer.gov/). RNA array dataset (GSE4290) (Sun et al., 2006) was downloaded from the NCBI/GEO database (https://www.ncbi.nlm.nih.gov/gds/). In this study, those data were used to perform gene expression analysis, clinical correlation analysis, and gene set enrichment analysis (GSEA) in LGG.

### Oncomine Database Analysis

As the largest oncogene database and integrated data-mining platform in the world, Oncomine (http://www.oncomine.org) is used to compare transcriptome data between tumors and corresponding normal tissues in different types of cancer (Rhodes et al., 2004). In this study, relevant data were obtained to evaluate the expression of GBP family genes in LGG. The *p*-value cutoff was 0.05. Statistical differences were determined by Student’s t-test.

### GEPIA Database Analysis

GEPIA (http://gepia.cancer-pku.cn/) is an interactive web application that analyzes RNA sequencing expression data for more than 9,000 tumors and 8,000 normal samples from The Cancer Genome Atlas (TCGA) and GTEx projects (Tang et al., 2017). In this study, we performed gene expression analysis and prognostic analysis of GBP genes both in pan-cancer and in LGG with GEPIA. Besides, gene correlation analysis was also evaluated with the Spearman correlation coefficient by GEPIA. The *p*-value cutoff was 0.05. Student’s t test was used to generate a *p*-value for expression, and a Kaplan–Meier curve was used for prognostic analysis.

### Tumor Immune Estimation Resource Database Analysis

TIMER (Tumor Immune Estimation Resource, https://cistrome.shinyapps.io/timer/) is a database designed for systematic analysis of immune cell infiltrates across diverse cancer types (Li et al., 2017). In our study, we evaluated the correlation between GBP gene levels and the infiltration of immune cells as well as the correlation among GBP gene expressions and marker gene expressions of the infiltration of immune cells and clinical outcome. Specifically, we analyzed the correlation between differentially expressed GBPs and macrophage polarity through the “correlation” module in the TIMER database. The results of Univariate Cox survival analysis in “Survival” module is shown in Figure 5E, and the results of Multivariate Cox survival analysis is shown in Table 3. The correlation map of differentially expressed GBPs and macrophage-related marker genes is shown in Figure 6. Spearman correlation coefficient was chosen for the correlation analysis.

### cBioPortal Database Analysis

CBioportal (http://www.cbioportal.org/) is an open platform for visualization, analysis, and download of multidimensional cancer genomics data (Gao et al., 2013). Based on the TCGA/LGG dataset (TCGA, provisional), we analyzed the genetic alterations and prognostic analysis of GBP genes in LGG.

### GSCALite

GSCALite is a user-friendly web server for dynamic analysis and visualization of gene sets in 32 cancer types from TCGA (Liu et al., 2018). In this study, GSCALite was used to analyze the miRNA regulatory network of GBP genes in LGG using the “TCGA KIRC” dataset.

### Search Tool for the Retrieval of Interacting Genes Database Analysis

The STRING (Search Tool for the Retrieval of Interacting Genes, https://string-db.org/) database aims to collect, score, and integrate both experimental as well as predicted protein–protein interaction (PPI) information and further achieve a comprehensive and objective global network, including direct (physical) as well as indirect (functional) interactions (Szklarczyk et al., 2019). In this study, we conducted a PPI network analysis of each GBP gene to explore the interactions of GBP genes.

### GeneMANIA Database Analysis

GeneMANIA (http://www.genemania.org) provides information for protein and genetic interactions, pathways, co-expression, co-localization, and protein domain similarity of submitted genes and helps researchers predict the functions behind gene sets by constructing a protein–protein interaction (PPI) network (Warde-Farley et al., 2010).

### Gene Set Enrichment Analysis

The gene set enrichment analysis (GSEA) was performed to identify significantly enriched groups of genes (Subramanian et al., 2005). In this study, the GSEA v4.0.3 software was applied to analyze biological pathway divergences between high and low GBP1/2/3/4 mRNA in the LGG expression profiles of TCGA data. The V7.0. Gene set in the gene set database and 1,000 for the number of permutations were selected for each analysis.

### Statistical Methods

In this study, SPSS 20.0 and GraphPad Prism 6.0 software were used for statistical analysis. The differential expression levels of GBPs were compared and analyzed by the Students’t-test. Survival curves were generated from Kaplan–Meier Plotter, and their differences are analyzed using log-rank test in GEPIA. Chi-square test was used to determine the correlation between expressions of GBPs and clinical parameters. The correlation between expressions of GBPs and immune infiltration level or other marker genes in LGG were evaluated by Spearman’s correlation and statistical significance. For GSEA, *p* < 0.05 and FDR (false discovery rate) *q* < 0.05 were considered as threshold values to estimate statistical significance.

## Results

### Guanylate-Binding Proteins 1/2/3/4 Were Upregulated in Lower-Grade Glioma Patients

**FIGURE 1:**
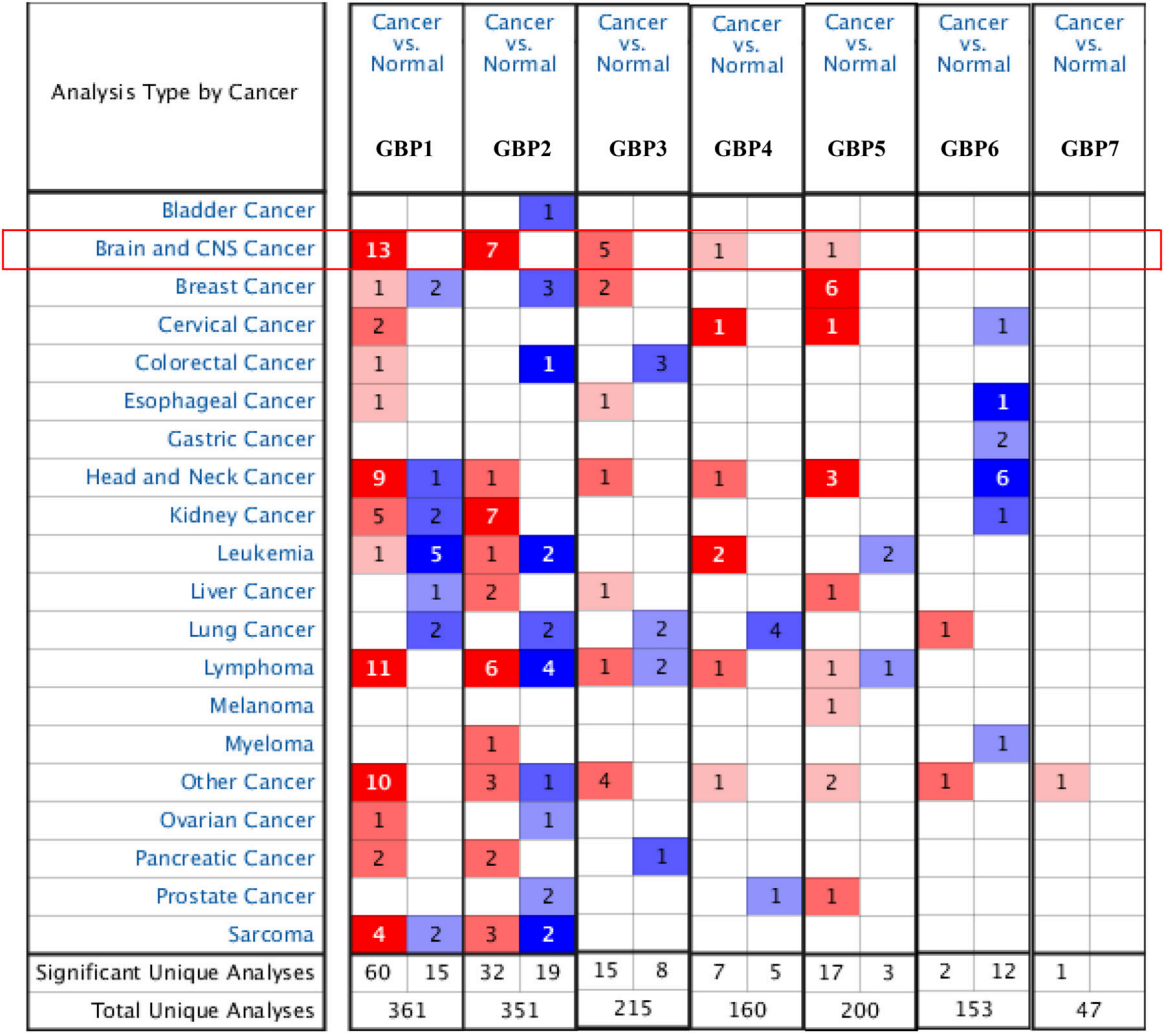
The expression levels of guanylate-binding proteins (GBPs) in different types of cancers (Oncomine). The expression levels of GBP1, 2, 3, 4, 5, 6, 7 in different types of cancers. Red, over-expression; blue, downregulated expression.

**FIGURE 2:**
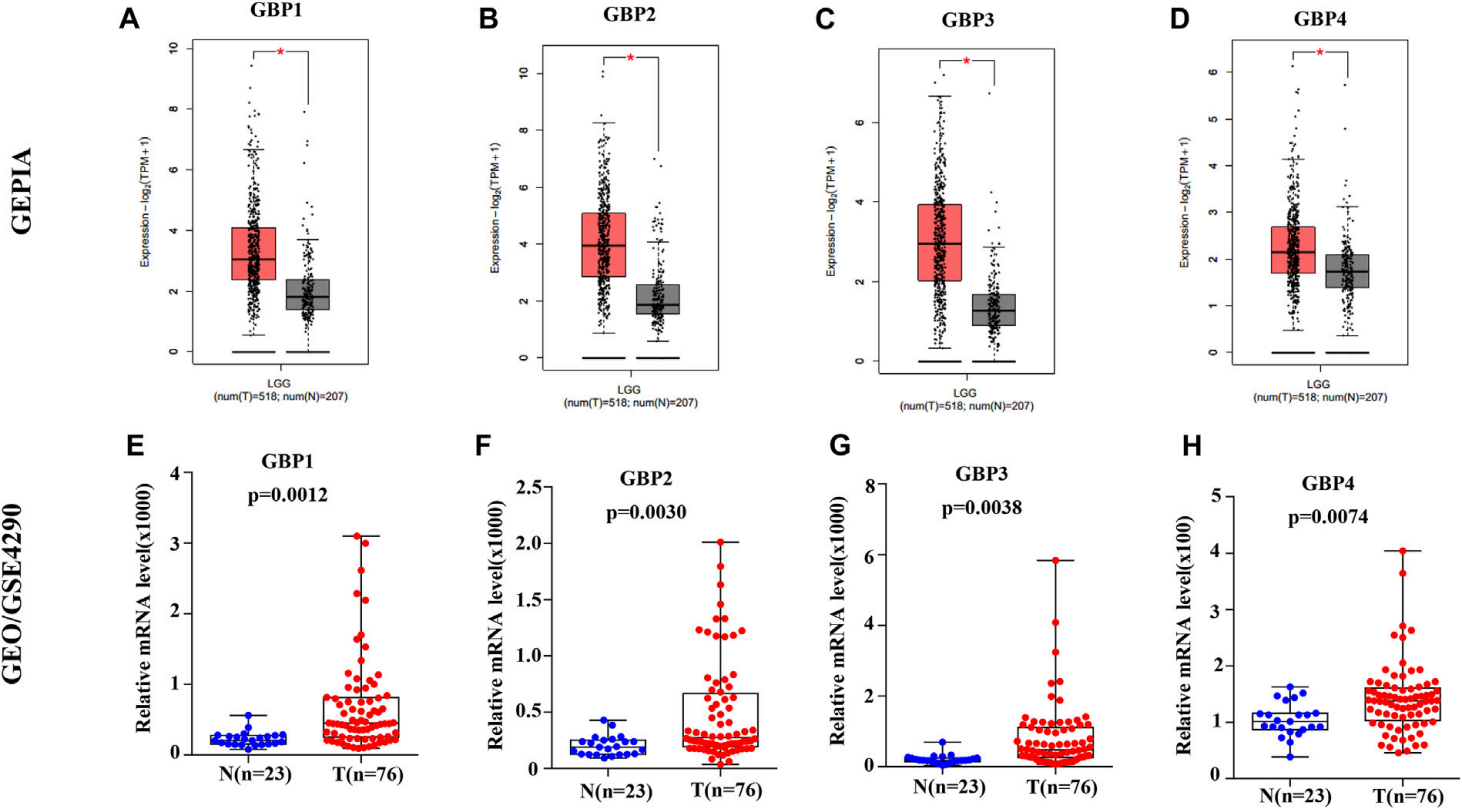
The expression levels of GBP1, 2, 3, 4 in lower-grade glioma (LGG) patients. The expression levels of **(A)** GBP1 **(B)** GBP2, **(C)** GBP3, and **(D)** GBP4 in LGG tissues vs normal tissues (GEPIA). The expression levels of **(E)** GBP1 **(F)** GBP2, **(G)** GBP3, and **(H)** GBP4 in LGG tissues vs normal tissues (GSE4290). *p < 0.05.

To explore the expression patterns of GBP family genes in brain and nervous system tumors, especially LGG, we first conducted a comprehensive analysis of the expression patterns of different GBP members using the Oncomine database. As shown in Figure 1, GBP1, 2, 3, 4, 5 were upregulated in the brain and nervous system tumors vs normal brain tissue, which was confirmed by data from 13, 7, 5, 1, and 1 datasets, respectively. Among them, the analysis results from the four datasets (Table 1) simultaneously confirmed that the expression of GBP1 and GBP2 was significantly increased in LGG vs normal brain tissue, and the results from two datasets and one dataset, respectively, demonstrated that the expression of GBP3 and GBP4 significantly increased in LGG. To further determine the expression differences of these four GBP genes, we selected the GEPIA database and one GEO dataset for verification. As shown in Figures 2A–H, the data from both GEPIA database and GSE4290 dataset demonstrated that GBP1, 2, 3, 4 were significantly upregulated in LGG.

**TABLE 1:** Significant changes of GBPs expression in different types of LGG tissues vs normal brain tissues (ONCOMINE).

Gene	Datasets	Type	Fold change	*p*-value	*t*-test
GBP1	Bredel Brain 2	Oligodendroglioma	2.019	0.006	3.717
		Anaplastic Oligoastrocytoma	3.026	0.007	3.501
	Sun Brain	Diffuse Astrocytoma	3.058	0.003	3.932
		Anaplastic Astrocytoma	3.000	1.85E-5	5.029
	French Brain	Anaplastic Oligoastrocytoma	5.428	0.004	4.677
	Rickman Brain	Astrocytoma	7.961	0.007	3.438
GBP2	Sun Brain	Anaplastic Astrocytoma	2.329	6.24E-5	4.567
	Bredel Brain 2	Anaplastic Oligoastrocytoma	4.096	6.53E-4	5.135
		Oligodendroglioma	2.920	0.009	3.354
	Rickman Brain	Astrocytoma	9.618	0.007	3.305
	French Brain	Anaplastic Oligoastrocytoma	3.235	0.019	3.474
GBP3	Sun Brain	Diffuse Astrocytoma	4.204	0.002	3.903
		Anaplastic Astrocytoma	3.706	6.09E-5	4.503
	French Brain	Anaplastic Oligodendroglioma	2.163	0.001	3.362
GBP4	Sun Brain	Diffuse Astrocytoma	2.813	0.018	2.576

### Prognostic Value of Guanylate-Binding Proteins 1/2/3/4 in Lower-Grade Glioma Patients

**FIGURE 3:**
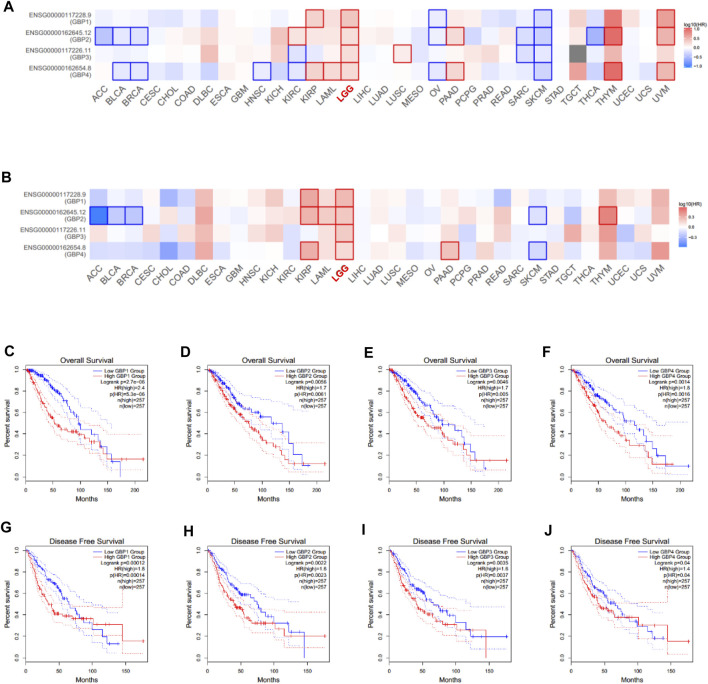
Prognostic value of GBP1/2/3/4 in LGG patients (GEPIA). Survival significance maps of GBP1, 2, 3, 4 in pan-cancer perspective showed the **(A)** over survival and **(B)** disease-free survival analysis results based on the Cox proportional hazard ratio (HR) (the red and blue blocks denote higher and lower risks, respectively; the rectangles with frames indicate significant unfavorable and favorable results). The overall survival curve of **(C)** GBP1 **(D)** GBP2, **(E)** GBP3, and **(F)** GBP4 in LGG patients. The disease-free survival curve of **(G)** GBP1 **(H)** GBP2, **(I)** GBP3, and **(J)** GBP4 in LGG patients.

We continued to explore the prognostic value of GBP1, 2, 3, 4 in LGG patients using GEPIA database by evaluating the effect of gene expression on overall survival and disease-free survival of tumor patients. The survival significance maps (Figures 3A,B) of pan-cancer based on the Cox proportional hazard ratio (HR) showed that GBP1, 2, 3, 4 had better prognostic value in LGG vs other tumor types, and highly expressed GBP1, 2, 3, 4 were all significantly unfavorable for both overall survival and disease-free survival of LGG patients. The Kaplan–Meier plots further demonstrated that LGG patients with highly expressed GBP1, 2, 3, 4 had shorter overall survival and disease-free survival time (Figures 3C–J).

### Correlations of Guanylate-Binding Proteins 1/2/3/4 With Clinicopathological characteristics in Lower-Grade Glioma

**FIGURE 4:**
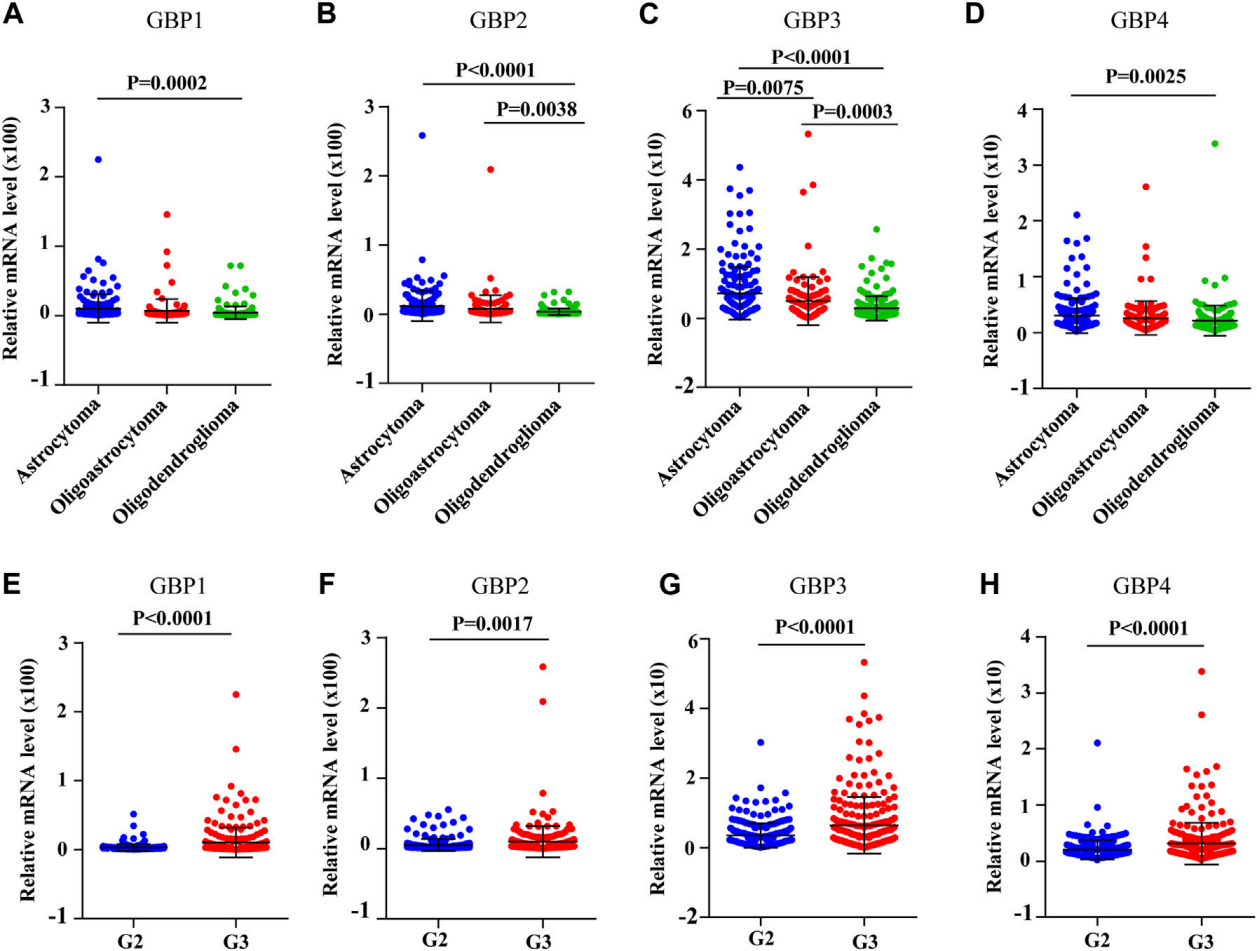
The expression levels of GBP1/2/3/4 in different histological subtypes and histological grades of LGG [The Cancer Genome Atlas (TCGA)]. The expression levels of **(A)** GBP1, **(B)** GBP2, **(C)** GBP3, and **(D)** GBP4 in the different histological subtypes of LGG. The expression levels of **(A)** GBP1, **(B)** GBP2, **(C)** GBP3, and **(D)** GBP4 in the different histological grades of LGG.

Next, we analyzed the correlations between GBP1, 2, 3, 4 expressions and clinicopathological characteristics. The expression data and clinical data of 509 LGG patients were extracted from the TCGA database, and clinical parameters mainly include age, sex, histological subtype, and tumor histological grade. Chi-square test was used to determine the correlation between GBP1, 2, 3, 4 expressions and clinical parameters. As shown in Table 2, the expressions of GBP1, 2, 3, 4 were significantly correlated with the histological subtype and histological grade of LGG patients, and only the expression of GBP4 had a correlation with the age and gender of the patient. Furthermore, we explored the expression differences of GBP1, 2, 3, 4 in different histological subtypes and histological grades of LGG patients. We found that the expressions of GBP1, 2, 3, 4 were significantly increased in astrocytoma vs oligodendroglioma, and GBP2, 3 were highly expressed in oligoastrocytoma vs oligodendroglioma (Figures 4A–D). Besides, we found that the expressions of GBP 1, 2, 3, 4 were all significantly increased in poor histological grade of LGG patients (Figures 4E–H), which was consistent with GBP 1, 2, 3, 4, which might be unfavorable factors for LGG patients.

**TABLE 2:** The correlation between GBP1/2/3/4 and clinicopathological parameters in LGG.

Parameters	N (*N* = 509)	GBP1			GBP2			GBP3			GBP4		
		Low	High	*p*	Low	High	*p*	Low	High	*p*	Low	High	*p*
Gender													
Female	228	120	108	0.267	116	112	0.692	114	114	0.968	125	103	0.045
		(56.2%)	(47.4%)		(50.9%)	(49.1%)		(50.0%)	(50.0%)		(54.8%)	(45.2%)	
Male	281	134	147		138	143		140	141		129	152	
		(47.7%)	(52.3%)		(49.1%)	(50.9%)		(49.8%)	(50.2%)		(45.9%)	(54.1%)	
Age													
<60	440	226	214	0.096	220	220	0.911	222	218	0.529	232	208	0.001
		(51.4%)	(48.6%)		(50.0%)	(50.0)%		(50.5%)	(49.5%)		(52.7%)	(47.3%)	
≥60	69	28	41		34	35		32	37		22	47	
		(40.6%)	(59.4%)		(49.3%)	(50.7%)		(46.4%)	(53.6%)		(31.9%)	(68.1%)	
Histological subtype													
Astrocytoma	192	59	133	0.000	53	139	0.000	59	133	0.000	82	110	0.012
		(30.7%)	(69.3%)		(27.6%)	(72.4%)		(54.7%)	(45.3%)		(42.7%)	(57.3%)	
Oligoastrocytoma	127	60	67		60	67		60	67		62	65	
		(47.2)%	(52.8%)		(47.2%)	(52.8%)		(55.9%)	(44.1%)		(48.8%)	(51.2%)	
Oligodendroglioma	190	135	55		141	49		135	55		110	80	
		(71.1%)	(28.9%)		(74.2%)	(25.8%)		(41.1%)	(58.9%)		(57.9%)	(42.1%)	
Grade													
G2	248	151	97	0.000	148	100	0.000	141	107	0.002	125	103	0.045
		(60.9%)	(39.1%)		(59.7%)	(40.3%)		(56.9%)	(43.1%)		(54.8%)	(45.2%)	
G3	261	103	158		106	155		113	148		129	152	
		(39.5%)	(60.5%)		(40.6%)	(59.4%)		(43.3%)	(56.7%)		(45.9%)	(54.1%)	

### Guanylate-Binding Protein P1/2/3/4 Expressions Were correlated With Immune cell Infiltration Levels in Lower-Grade Glioma Patients

**FIGURE 5:**
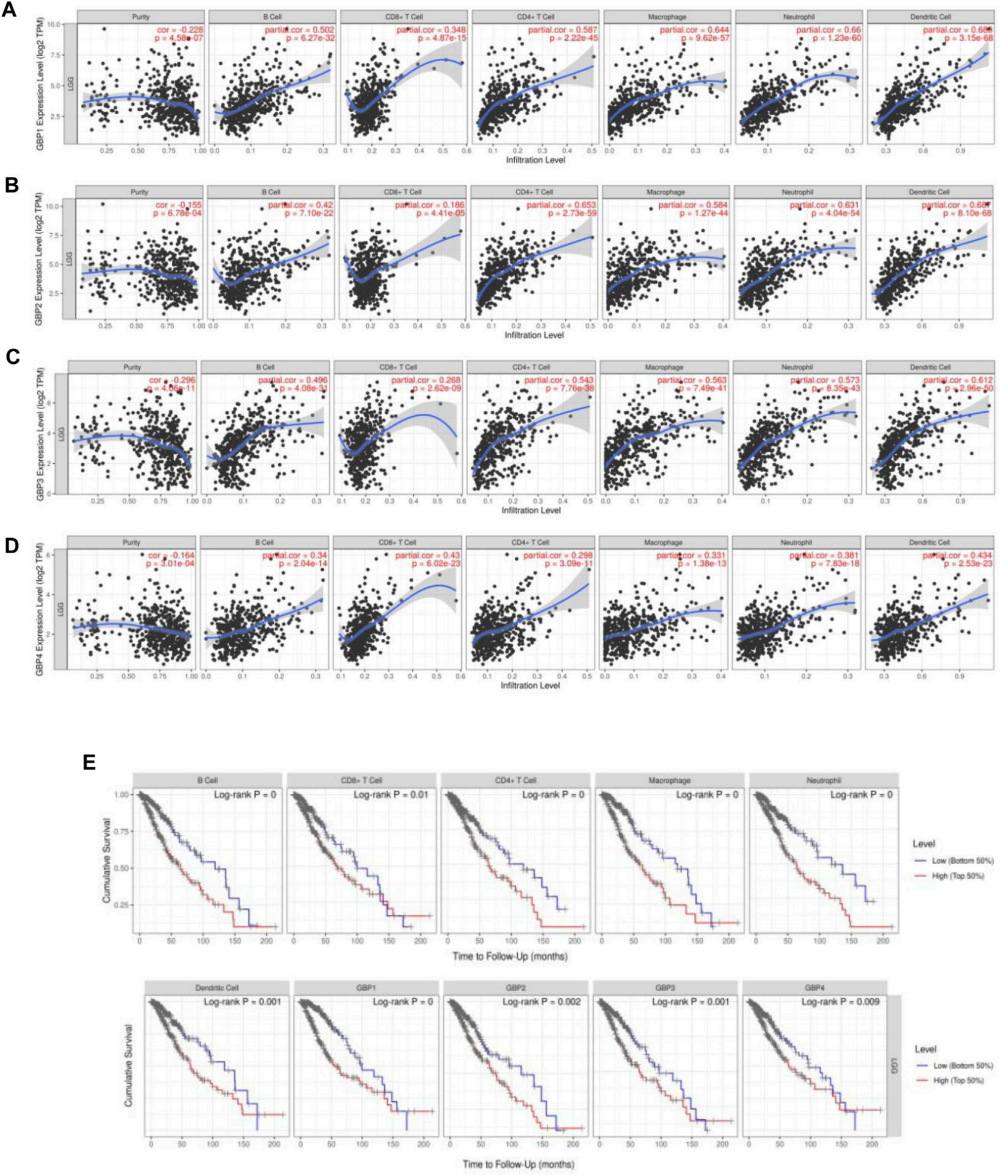
GBP1/2/3/4 expressions were correlated with immune cell infiltration levels in LGG patients [Tumor Immune Estimation Resource (TIMER)]. The correlation between the abundance of immune cells and the expression of **(A)** GBP1, **(B)** GBP2, **(C)** GBP3, and **(D)** GBP4 in LGG. **(E)** Kaplan–Meier plots of different immune-infiltrating cells and GBP1, 2, 3, 4 in LGG.

Immune cell infiltration in the tumor microenvironment is an important factor affecting tumor progression and prognosis of cancer patients (Domingues et al., 2016). In order to explore whether GBP1, 2, 3, 4 regulates the level of infiltrating immune cells in the LGG microenvironment, we analyzed the correlation of the expressions of GBP 1, 2, 3, 4 with immune infiltrating cells based on the TIME database (Figures 5A–D). Interestingly, we found that the expressions of GBP1, 2, 3, 4 were strongly and positively correlated with these immune-infiltrating cells, including B cells, CD8^+^ T cells, CD4^+^ T cells, macrophages, neutrophils, and dendritic cells. Furthermore, we explored the effects of six immune cells and GBP 1, 2, 3, 4 expressions on the prognosis of LGG patients. Univariate Cox survival analysis showed that the high infiltration levels of six types of immune cells and the high expressions of GBP 1, 2, 3, 4 indicated poor prognosis of LGG patients (Figures 5E). Multivariate Cox survival analysis showed that macrophages, GBP1, and GBP2 were independent prognostic indicators for LGG patients (Table 3). These findings indicated that GBP1, 2, 3, 4 may potentially regulate the level of immune cell infiltration in LGG, and a high level of immune cell infiltration is not conducive to patient survival.

**TABLE 3:** The cox proportional hazard model of GBP1/2/3/4 and six tumor-infiltrating immune cells in KIRC (TIMER).

	Coef	HR	95%CI_l	95%CI_u	*p*-value	Sig
B_cell	4.646	104.126	0.339	31971.731	0.112	
CD8+_T cell	4.433	84.143	0.063	113191.980	0.228	
CD4+_T cell	−1.125	0.325	0.000	1180.514	0.788	
Macrophage	6.067	431.528	6.532	28506.295	0.005	**
Neutrophil	−8.021	0.000	0.000	1.114	0.053	
dendritic cell	−0.573	0.564	0.013	25.001	0.767	
GBP1	0.659	1.934	1.498	2.497	0.000	***
GBP2	−0.015	0.799	0.657	0.971	0.024	*
GBP3	−0.031	0.985	0.819	1.184	0.872	
GBP4	−0.150	0.861	0.647	1.146	0.304	

### Correlation Analysis Between Guanylate-Binding Protein 1/2/3/4 and Immune Markers in Lower-Grade Glioma Patients

**FIGURE 6:**
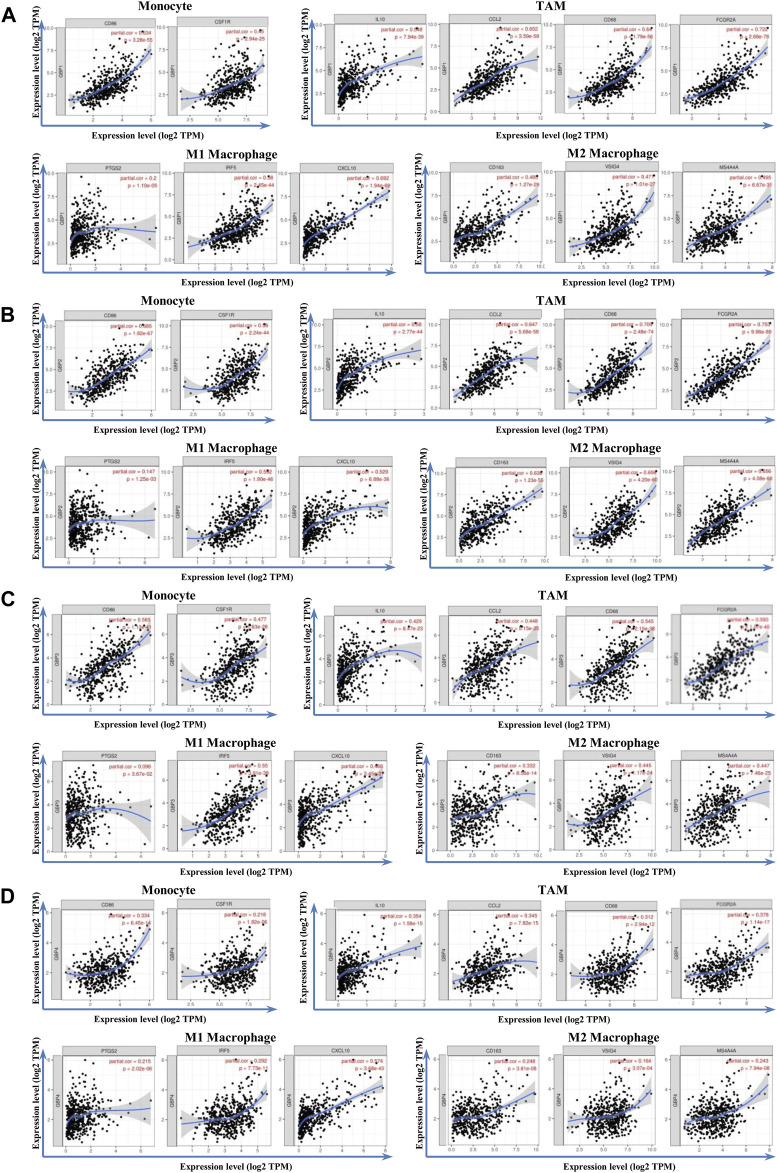
Correlation analysis between GBP1/2/3/4 and immune markers in LGG patients. The correlation between the expression of GBP1/2/3/4 and monocytes (gene markers: CD86 and CD115), TAM (gene markers: IL10, CCL2, CD68, and FCGR2A), M1 macrophage (gene markers: PTGS2, IRF5, and CCL10), and M2 macrophage (gene markers: CD163, VSIG4, and MS4A4A) infiltration levels was assessed. Scatterplots of correlations between monocytes, TAM, M1 macrophage, M2 macrophage, and the expressions of **(A)** GBP1 **(B)** GBP2, **(C)** GBP3, and **(D)** GBP4 in LGG

To further clarify the relationship of GBP 1, 2, 3, 4 with immune infiltration, we analyzed the correlation between the expressions of GBP 1, 2, 3, 4 and gene markers of a variety of immune cells (Table 4). We found that GBP1 had a significant correlation with most of the gene markers of infiltrating immune cells, excluding one gene marker (STAT4) of T-helper 1 (Th1) cell, two gene markers (FOXP3 and STAT5B) of regulatory T cell (Treg), and two gene markers (KIR2DL1 and KIR3DL3) of natural killer cell. GBP2 had a strong correlation with almost all markers of infiltrating immune cells, except for the two gene markers (KIR2DL1 and KIR3DL3) of natural killer cell. GBP3 also showed a significant correlation with most markers of infiltrating immune cells, excluding two markers (FOXP3 and STAT5B) of Treg and four markers (KIR2DL1, KIR3DL1, KIR3DL3, and KIR2DS4) of natural killer cell. GBP4 was significantly connected with most markers of infiltrating immune cells, excluding one marker (STAT5B) of Treg, one marker (KIR3DL3) of natural killer cell, and one marker (LAG3) of exhausted T cell. Especially, GBP1, 2, 3, 4 expressions had a strong correlation with the gene markers of infiltrating monocytes, TAM, M1, and M2 macrophages in LGG (Table 4 and Figure 6), which also had been verified in the GEPIA database (Table 5). This indicated that GBP1, 2, 3, 4 may be involved in regulating macrophage polarity in LGG.

**TABLE 4:** Correlation analysis between GBP1/2/3/4 and related markers of immune cells in LGG.

Description	Gene marker	GBP1		GBP2		GBP3		GBP4	
		Cor	*p*	Cor	*p*	Cor	*p*	Cor	*p*
Monocyte	CD86	0.634	***	0.685	***	0.565	***	0.334	***
	CD115 (CSF1R)	0.450	***	0.580	***	0.477	***	0.216	***
TAM	IL10	0.548	***	0.580	***	0.429	***	0.354	***
	CCL2	0.652	***	0.647	***	0.448	***	0.345	***
	CD68	0.640	***	0.709	***	0.545	***	0.312	***
	FCGR2A	0.722	***	0.753	***	0.593	***	0.378	***
M1 Macrophage	PTGS2	0.200	***	0.147	**	0.096	*	0.215	***
	IRF5	0.580	***	0.592	***	0.550	***	0.292	***
	CXCL10	0.692	***	0.529	***	0.466	***	0.574	***
M2 Macrophage	CD163	0.485	***	0.636	***	0.332	***	0.248	***
	VSIG4	0.471	***	0.656	***	0.445	***	0.164	***
	MS4A4A	0.495	***	0.656	***	0.447	***	0.243	***
DCs	ITGAX	0.505	***	0.451	***	0.447	***	0.229	***
	CD1C	0.357	***	0.440	***	0.266	***	0.306	***
	NRP1	0.323	***	0.287	***	0.244	***	0.471	***
	THBD	0.378	***	0.389	***	0.216	***	0.350	***
Neutrophils	CCR7	0.412	***	0.313	***	0.261	***	0.356	***
	ITGAM	0.556	***	0.598	***	0.547	***	0.315	***
	CD59	0.266	***	0.253	***	0.132	**	0.233	***
Th1	STAT4	−0.087	0.059	−0.282	***	−0.166	***	0.141	**
	TBX21	0.358	***	0.282	***	0.25	***	0.313	***
	CD4	0.608	***	0.707	***	0.539	***	0.370	***
Th2	CXCR4	0.531	***	0.375	***	0.371	***	0.231	***
	CCR4	0.433	***	0.324	***	0.312	***	0.429	***
	CCR8	0.204	***	0.143	**	0.144	**	0.149	**
Treg	FOXP3	−0.071	0.120	−0.289	***	−0.081	0.077	0.110	*
	STAT5B	0.020	0.662	0.114	*	0.074	0.107	0.063	0.171
	TGFB1	0.563	***	0.642	***	0.542	***	0.202	***
Natural killer cell	KIR2DL1	0.053	0.246	0.035	0.448	−0.004	0.936	0.104	*
	KIR2DL3	0.174	***	0.217	***	0.118	*	0.171	***
	KIR3DL1	0.103	*	0.103	*	−0.029	0.521	0.189	***
	KIR3DL2	0.189	***	0.175	***	0.183	***	0.179	***
	KIR3DL3	−0.012	0.797	0.019	0.672	0.022	0.630	−0.054	0.241
	KIR2DS4	0.172	***	0.198	***	0.050	0.276	0.244	***
T cell exhaustion	PDCD1(PD-1)	0.586	***	0.531	***	0.401	***	0.283	***
	CTLA4	0.318	***	0.288	***	0.236	***	0.302	***
	TIM-3 (HAVCR2)	0.649	***	0.674	***	0.587	***	0.334	***
	GZMB	0.366	***	0.276	***	0.171	***	0.405	***
	LAG3	0.231	***	0.326	***	0.185	***	0.049	0.287

**TABLE 5:** Correlation analysis between GBP1/2/3/4 and related markers of immune cells in LGG (GEPIA).

Description	Gene marker	GBP1		GBP2		GBP3		GBP4	
		Cor	*p*	Cor	*P*	Cor	*p*	Cor	*p*
Monocyte	CD86	0.67	***	0.67	***	0.63	***	0.40	***
	CD115 (CSF1R)	0.52	***	0.57	***	0.56	***	0.27	***
TAM	IL10	0.58	***	0.56	***	0.49	***	0.40	***
	CCL2	0.68	***	0.66	***	0.48	***	0.40	***
	CD68	0.67	***	0.69	***	0.61	***	0.37	***
	FCGR2A	0.76	***	0.76	***	0.64	***	0.44	***
M1 Macrophage	PTGS2	0.27	***	0.17	***	0.18	***	0.20	***
	IRF5	0.62	***	0.59	***	0.60	***	0.39	***
	CXCL10	0.70	***	0.54	***	0.49	***	0.63	***
M2 Macrophage	CD163	0.50	***	0.66	***	0.33	***	0.27	***
	VSIG4	0.54	***	0.66	***	0.51	***	0.22	***
	MS4A4A	0.53	***	0.67	***	0.47	***	0.30	***

### Gene Alterations, co-expression, Interaction Network Analysis of Guanylate-Binding Protein 1/2/3/4 in Lower-Grade Glioma

**FIGURE 7:**
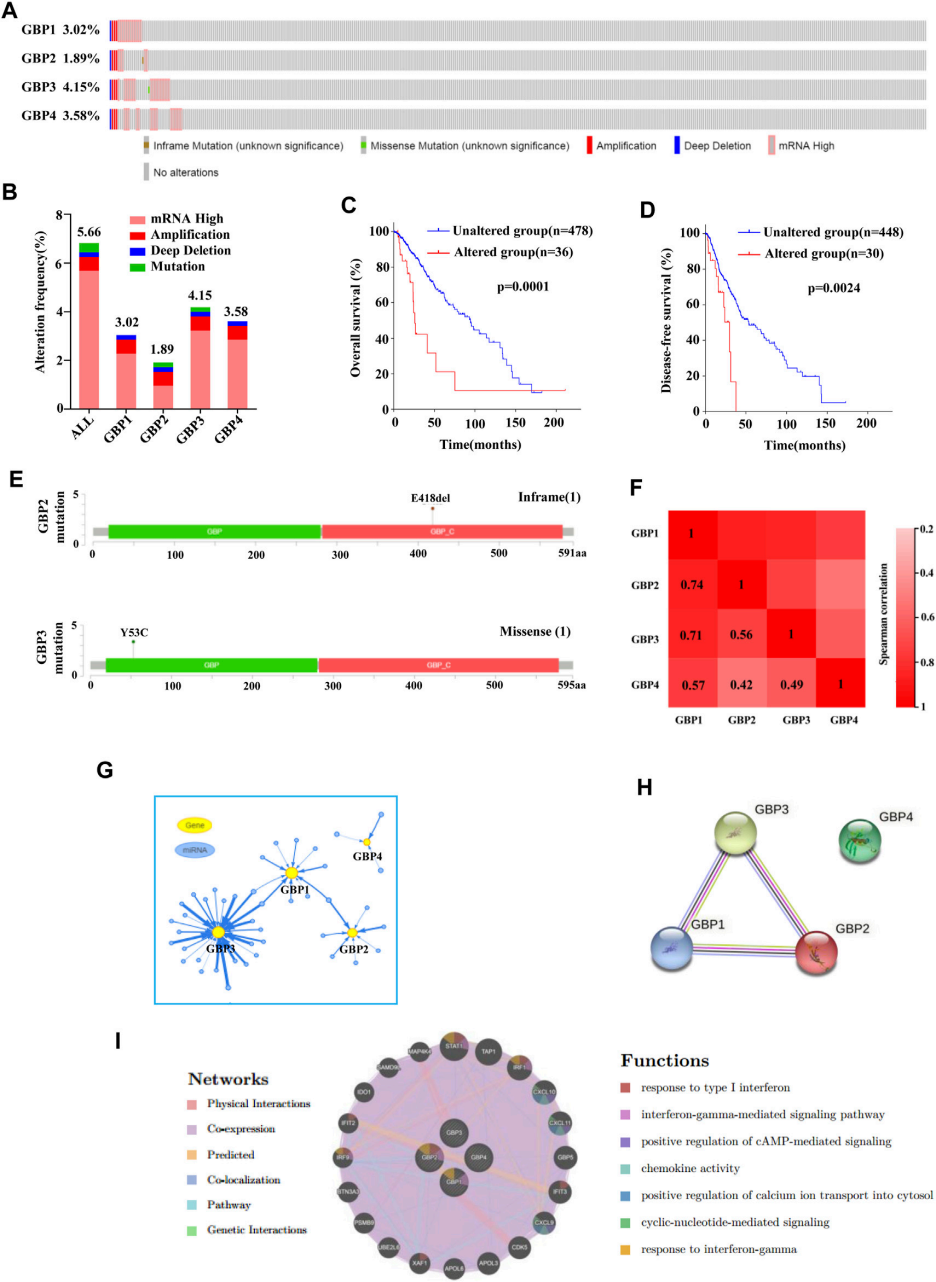
Gene alterations, co-expression, interaction network analysis of GBP1, 2, 3, 4 in LGG. **(A, B)** Summary of gene alterations of GBP1, 2, 3, 4 in LGG (cBioPortal). **(C, D)** Overall survival and disease-free survival analysis results of GBP1, 2, 3, 4 gene alterations (cBioPortal). **(E)** The mutations of GBP2 and 3 were plotted (cBioPortal). **(G)** miRNA network of GBP1, 2, 3, 4 in LGG (GSCALite). **(H)** Protein–protein interaction network of GBP1, 2, 3, 4 (STRING). **(I)** The interaction network and function prediction of GBP1, 2, 3, 4 (GeneMANIA).

Then we focused on the gene alterations of GBP1, 2, 3, 4 in LGG using the cBioPortal platform. A total of 518 LGG patients were selected for this analysis. The genetic alteration frequency of GBP1, 2, 3, 4 in LGG, including amplification, high mRNA, deep deletion, and mutation, was 3.02, 1.89, 4.15, 3.58%, respectively (Figure 7A). The total genetic alteration frequency of GBP1, 2, 3, 4 was 5.66%, and high mRNA was the most common type of gene alteration in these samples (Figure 7B). We further evaluated the impact of genetic alteration of GBP 1, 2, 3, 4 on patient survival and found that LGG patients with the genetic alteration of GBP1, 2, 3, 4 have shorter overall survival and disease-free survival time (Figures 7C,D). We also found there was an inframe mutation in GBP2, and a missense mutation in GBP3 (Figure 7E). We continue to explore the co-expression and interaction network of GBP 1, 2, 3, 4. We found that there was a strong expression correlation among GBP1, 2, 3 and 4 (Figure 7F), and the functions of GBP1, 2, 3 and 4 were potentially regulated by different miRNAs (Figure 7G). Also, there was a close interaction relationship between GBP1, 2, and 3 (Figure 7H), and the functions of GBP1, 2, 3, 4 are mainly related to cellular response to type I interferon, interferon-gamma-mediated signaling pathway, chemokine activity, positive regulation of cAMP-mediated signaling, etc (Figure 7I).

### Pathway Enrichment Analysis of Guanylate-Binding Protein 1/2/3/4 in Lower-Grade Glioma

**FIGURE 8:**
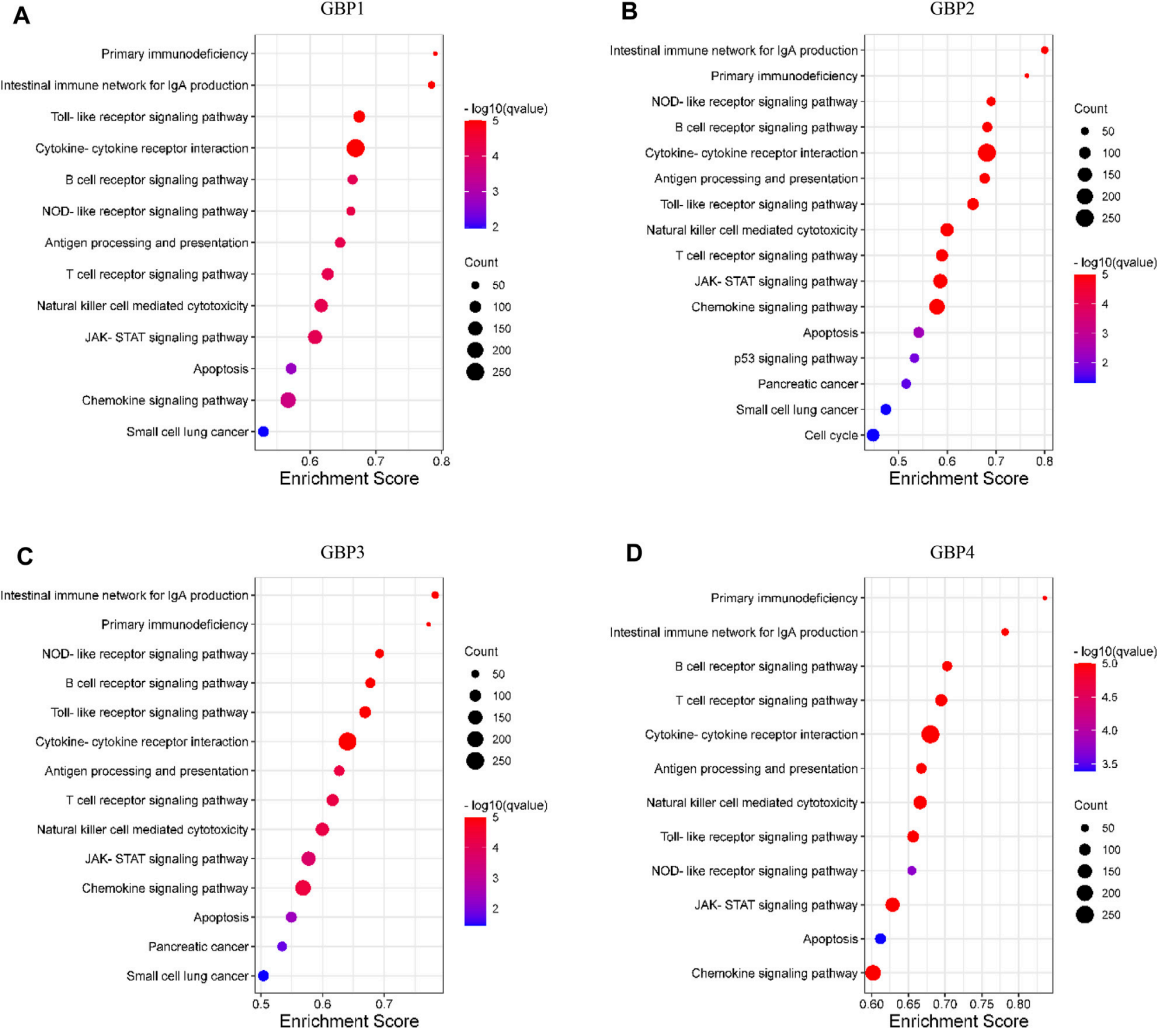
Pathway enrichment analysis of GBP1, 2, 3, 4 in LGG. The bubble diagram displayed the gene set enrichment analysis (GSEA) results in the phenotype high group of **(A)** GBP1 **(B)** GBP2, **(C)** GBP3, and **(D)** GBP4 in LGG. The nominal *p*-value (NOM *p* < 0.05) and false discovery rate (FDR q < 0.05) were used to select significantly enriched gene sets.

GSEA is used to explore the signaling pathways that are potentially regulated by GBP1, 2, 3, 4 in LGG. We divided the samples into the high-expression group and the low-expression group based on the mean value. Pathways with higher frequency enriched in phenotype high of GBP1, 2, 3, 4 are presented in Figures 8A–D. We found that functions of GBP1, 2, 3, 4 were closely linked: 1) They were all involved in regulating immune-related signaling pathways, such as intestinal immune network for IgA production, primary immunodeficiency, B/T cell receptor signaling pathway, natural killer cell-mediated cytotoxicity, etc. 2) They were all closely related to cancer and participated in the regulation of cancer-related signaling pathways, such as JAK-STAT signaling pathway, apoptosis, etc. 3) They also regulated Toll-like receptor pathway signaling, NOD-like receptor signaling pathway, and chemokine signaling pathway. Together, these results indicated that GBP1, 2, 3, 4 had the potential to become therapeutic targets in LGG.

## Discussion

Previous studies have identified a family of IFN-inducible GTPases, namely, guanylate-binding proteins (GBPs), as a major nexus of IFN-driven complex homeostatic defense networks, which function in host defense to viral, bacterial, and protozoan pathogens (Tretina et al., 2019). In recent years, an increasing number of studies have also confirmed that GBPs are not only involved in regulating host immune defense but also closely related to tumor development and metastasis (Mustafa et al., 2018; Zhao et al., 2019; Yu et al., 2020), and some GBPs, such as GBP1 and GBP2, have shown good prognostic value in certain tumors, for example, breast, oral and colorectal cancer (Yu et al., 2011; Godoy et al., 2014; Wang et al., 2016). Elevated GBP1 expression has also been shown to be associated with chemotherapy resistance in lung, breast, and ovarian cancer (Duan et al., 2006; Fekete and Győrffy, 2019; Cheng et al., 2020). However, the biological function and prognostic value of individual GBP in LGG remain elusive.

By analysis and verification through multiple public data platforms, we found that GBP1, 2, 3, 4 were significantly upregulated in LGG tissues vs normal brain tissue. Consistently, we further found that highly expressed GBP1, 2, 3, 4 were all significantly unfavorable for both overall survival and disease-free survival of LGG patients, suggesting the potential of GBP1, 2, 3, 4 as prognostic markers in LGG. To further explore the clinical significance of GBPs, we analyzed the correlation between their expressions and the clinical parameters of LGG patients, and we found that the expressions of GBP 1, 2, 3, and 4 were significantly associated with tumor histological grade of LGG. As the tumor histological grade increased, the expressions of GBP 1, 2, 3, and 4 significantly increased. This was consistent with GBP 1, 2, 3, 4, which might be unfavorable factors for LGG patients. Besides, the expressions of GBP1, 2, 3, 4 were significantly correlated with the histological subtype of LGG, and the expression of GBP4 also had a correlation with the age and gender of the patient.

Infiltrating immune cells in the tumor microenvironment, mainly including tumor-infiltrating lymphocytes (B cells, CD8^+^ T cells, and CD4^+^ T cells) and other immune cells (macrophages, neutrophils, and dendritic cells), have become the focus of current tumor research. Studies have shown that immune-infiltrating cells play an indispensable function in the tumor microenvironment as a double-edged sword to promote or inhibit tumor cell progression (Fridman et al., 2012). On the one hand, immune infiltrating cells play an anti-tumor effect by monitoring and destroying cancer cells (Morvan and Lanier, 2016). On the other hand, studies have shown that cancer cells can evade the surveillance of immune-infiltrating cells through a variety of mechanisms or further manipulate these immune infiltrating cells to create a microenvironment that promotes tumor progression (Mantovani et al., 2008). The dual effect of immune-infiltrating cells on tumor cells may depend on the type of immune cells, the state of immune cells, and the microenvironment of different tumors (Domingues et al., 2016). In our study, we found that the expressions of GBP1, 2, 3, 4 were significantly and positively correlated with the levels of all the six immune-infiltrating cells evaluated. Univariate analysis further showed that high expressions of GBP1, 2, 3, 4 and high levels of six immune-infiltrating cells were poor prognostic factors for LGG patients. Multivariate analysis showed that GBP1, GBP2, and macrophage infiltration are independent prognostic factors for LGG patients. The correlation between the expression of GBP 1, 2, 3, 4 and the expressions of other immune cell marker genes were assessed, further confirming the close connection between GBP 1, 2, 3, 4 and tumor immune-infiltrating cells. In particular, the expression of GBP 1, 2, 3, 4 are significantly and positively correlated with the marker genes of monocytes, TAM, M1, and M2 macrophages, indicating that GBP 1, 2, 3, 4 may be involved in regulating the polarity of macrophages. However, how GBP 1, 2, 3, 4 participate in the regulation of tumor immune-infiltrating cells requires further research to clarify.

Then, we focused on the genetic alterations of GBP1, 2, 3 and 4 in LGG, and we found that mRNA high is the most common type of genetic alterations in all LGG patient samples with genetic alterations. The genetic alterations of GBP1, 2, 3 and 4 indicated a poor prognosis of LGG patients. We also found that there was an inframe mutation in GBP2, and a missense mutation in GBP3. Co-expression and interaction network analysis further revealed the close functional connection among them.

Finally, we used the GSEA method to explore the signaling pathways that may be potentially regulated by GBP1, 2, 3, and 4 in LGG. We found that GBP1, 2, 3, 4 are closely related to immune-related signaling pathways, such as intestinal immune network for IgA production, primary immunodeficiency, B/T cell receptor signaling pathway, natural killer cell-mediated cytotoxicity, etc., which was consistent with the association between GBP1, 2, 3, 4 and immune cell infiltration that we explored above. Besides, we found that they also potentially regulated Toll-like receptor pathway signaling, NOD-like receptor signaling pathway, and chemokine signaling pathway. Together, these results indicated that GBP1, 2, 3, 4 had the potential to become therapeutic targets in LGG.

Our study still has some limitations. The analysis of gene transcription levels based on public data platforms cannot fully reflect the changes in protein levels. Therefore, experiments *in vivo* and *in vitro* are needed to verify our findings and further promote the understanding of GBPs in LGG. Despite these limitations, our study may help guide further investigation of GBPs in LGG.

In conclusion, we systematically and comprehensively analyzed the expression pattern, prognostic value, correlation with clinical parameters, immune infiltration relevance of GBPs in LGG, and further explored their potential regulatory signaling pathways. Our results indicated that GBP1, 2, 3, 4 were potential biomarkers that can be used to predict prognosis and tumor immune infiltration of LGG patients. We hope that our results can help clinicians better predict the survival of LGG patients or choose appropriate treatment methods or therapeutic drugs, thereby improving the survival prognosis of cancer patients.

## Data Availability

The original contributions presented in the study are included in the article/supplementary material, further inquiries can be directed to the corresponding authors.

## References

[B1] Brat D. J. Verhaak R. G. Aldape K. D. Yung W. K. Salama S. R. Cooper L. A. (2015). Comprehensive, Integrative Genomic Analysis of Diffuse Lower-Grade Gliomas. N. Engl. J. Med. 372 (26), 2481–2498. 10.1056/NEJMoa1402121 26061751 PMC4530011

[B2] Cheng L. Gou L. Wei T. Zhang J. (2020). GBP1 Promotes Erlotinib Resistance via PGK1 Activated EMT Signaling in Non Small Cell Lung Cancer. Int. J. Oncol. 57 (3), 858–870. 10.3892/ijo.2020.5086 32582960

[B3] De Donato M. Mariani M. Petrella L. Martinelli E. Zannoni G. F. Vellone V. (2012). Class III β-tubulin and the Cytoskeletal Gateway for Drug Resistance in Ovarian Cancer. J. Cel. Physiol. 227 (3), 1034–1041. 10.1002/jcp.22813 21520077

[B4] Domingues P. González-Tablas M. Otero Á. Pascual D. Miranda D. Ruiz L. (2016). Tumor Infiltrating Immune Cells in Gliomas and Meningiomas. Brain Behav. Immun. 53, 1–15. 10.1016/j.bbi.2015.07.019 26216710

[B5] Duan Z. Foster R. Brakora K. A. Yusuf R. Z. Seiden M. V. (2006). GBP1 Overexpression Is Associated with a Paclitaxel Resistance Phenotype. Cancer Chemother. Pharmacol. 57 (1), 25–33. 10.1007/s00280-005-0026-3 16028104

[B6] Fekete J. T. Győrffy B. (2019). ROCplot.org: Validating Predictive Biomarkers of Chemotherapy/hormonal therapy/anti‐HER2 Therapy Using Transcriptomic Data of 3,104 Breast Cancer Patients. Int. J. Cancer 145 (11), 3140–3151. 10.1002/ijc.32369 31020993

[B7] Fridman W. H. Pagès F. Sautès-Fridman C. Galon J. (2012). The Immune Contexture in Human Tumours: Impact on Clinical Outcome. Nat. Rev. Cancer 12 (4), 298–306. 10.1038/nrc3245 22419253

[B8] Gao J. Aksoy B. A. Dogrusoz U. Dresdner G. Gross B. Sumer S. O. (2013). Integrative Analysis of Complex Cancer Genomics and Clinical Profiles Using the cBioPortal. Sci. Signal. 6 (269), pl1. 10.1126/scisignal.2004088 23550210 PMC4160307

[B9] Ghosh A. Praefcke G. J. K. Renault L. Wittinghofer A. Herrmann C. (2006). How Guanylate-Binding Proteins Achieve Assembly-Stimulated Processive Cleavage of GTP to GMP. Nature 440 (7080), 101–104. 10.1038/nature04510 16511497

[B10] Godoy P. Cadenas C. Hellwig B. Marchan R. Stewart J. Reif R. (2014). Interferon-inducible Guanylate Binding Protein (GBP2) Is Associated with Better Prognosis in Breast Cancer and Indicates an Efficient T Cell Response. Breast Cancer 21 (4), 491–499. 10.1007/s12282-012-0404-8 23001506

[B11] Honkala A. T. Tailor D. Malhotra S. V. (2019). Guanylate-Binding Protein 1: An Emerging Target in Inflammation and Cancer. Front. Immunol. 10, 3139. 10.3389/fimmu.2019.03139 32117203 PMC7025589

[B12] Jang B.-S. Kim I. A. (2018). A Radiosensitivity Gene Signature and PD-L1 Predict the Clinical Outcomes of Patients with Lower Grade Glioma in TCGA. Radiother. Oncol. 128 (2), 245–253. 10.1016/j.radonc.2018.05.003 29784449

[B13] Ji X. Zhu H. Dai X. Xi Y. Sheng Y. Gao C. (2019). Overexpression of GBP1 Predicts Poor Prognosis and Promotes Tumor Growth in Human Glioblastoma Multiforme. CBM 25 (3), 275–290. 10.3233/CBM-171177 PMC13082430 29991124

[B14] Li T. Fan J. Wang B. Traugh N. Chen Q. Liu J. S. (2017). TIMER: A Web Server for Comprehensive Analysis of Tumor-Infiltrating Immune Cells. Cancer Res. 77 (21), e108–e110. 10.1158/0008-5472.CAN-17-0307 29092952 PMC6042652

[B15] Lipnik K. Naschberger E. Gonin-Laurent N. Kodajova P. Petznek H. Rungaldier S. (2010). Interferon γ-Induced Human Guanylate Binding Protein 1 Inhibits Mammary Tumor Growth in Mice. Mol. Med. 16 (5-6), 177–187. 10.2119/molmed.2009.00172 20454519 PMC2864808

[B16] Liu B. Liu J. Liu K. Huang H. Li Y. Hu X. (2019). A Prognostic Signature of Five Pseudogenes for Predicting Lower-Grade Gliomas. Biomed. Pharmacother. 117, 109116. 10.1016/j.biopha.2019.109116 31247469

[B17] Liu C.-J. Hu F.-F. Xia M.-X. Han L. Zhang Q. Guo A.-Y. (2018). GSCALite: a Web Server for Gene Set Cancer Analysis. Bioinformatics 34 (21), 3771–3772. 10.1093/bioinformatics/bty411 29790900

[B18] Mantovani A. Romero P. Palucka A. K. Marincola F. M. (2008). Tumour Immunity: Effector Response to Tumour and Role of the Microenvironment. Lancet 371 (9614), 771–783. 10.1016/S0140-6736(08)60241-X 18275997

[B19] Mazurowski M. A. Clark K. Czarnek N. M. Shamsesfandabadi P. Peters K. B. Saha A. (2017). Radiogenomics of Lower-Grade Glioma: Algorithmically-Assessed Tumor Shape Is Associated with Tumor Genomic Subtypes and Patient Outcomes in a Multi-Institutional Study with the Cancer Genome Atlas Data. J. Neurooncol. 133 (1), 27–35. 10.1007/s11060-017-2420-1 28470431

[B20] Morvan M. G. Lanier L. L. (2016). NK Cells and Cancer: You Can Teach Innate Cells New Tricks. Nat. Rev. Cancer 16 (1), 7–19. 10.1038/nrc.2015.5 26694935

[B21] Mustafa D. A. M. Pedrosa R. M. S. M. Smid M. van der Weiden M. de Weerd V. Nigg A. L. (2018). T Lymphocytes Facilitate Brain Metastasis of Breast Cancer by Inducing Guanylate-Binding Protein 1 Expression. Acta Neuropathol. 135 (4), 581–599. 10.1007/s00401-018-1806-2 29350274 PMC5978929

[B22] Naschberger E. Croner R. S. Merkel S. Dimmler A. Tripal P. Amann K. U. (2008). Angiostatic Immune Reaction in Colorectal Carcinoma: Impact on Survival and Perspectives for Antiangiogenic Therapy. Int. J. Cancer 123 (9), 2120–2129. 10.1002/ijc.23764 18697200

[B23] Rhodes D. R. Yu J. Shanker K. Deshpande N. Varambally R. Ghosh D. (2004). ONCOMINE: a Cancer Microarray Database and Integrated Data-Mining Platform. Neoplasia 6 (1), 1–6. 10.1016/s1476-5586(04)80047-2 15068665 PMC1635162

[B24] Simonelli M. Persico P. Perrino M. Zucali P. A. Navarria P. Pessina F. (2018). Checkpoint Inhibitors as Treatment for Malignant Gliomas: "A Long Way to the Top". Cancer Treat. Rev. 69, 121–131. 10.1016/j.ctrv.2018.06.016 29966936

[B25] Subramanian A. Tamayo P. Mootha V. K. Mukherjee S. Ebert B. L. Gillette M. A. (2005). Gene Set Enrichment Analysis: a Knowledge-Based Approach for Interpreting Genome-wide Expression Profiles. Proc. Natl. Acad. Sci. 102 (43), 15545–15550. 10.1073/pnas.0506580102 16199517 PMC1239896

[B26] Sun L. Hui A.-M. Su Q. Vortmeyer A. Kotliarov Y. Pastorino S. (2006). Neuronal and Glioma-Derived Stem Cell Factor Induces Angiogenesis within the Brain. Cancer cell 9 (4), 287–300. 10.1016/j.ccr.2006.03.003 16616334

[B27] Szklarczyk D. Gable A. L. Lyon D. Junge A. Wyder S. Huerta-Cepas J. (2019). STRING V11: Protein-Protein Association Networks with Increased Coverage, Supporting Functional Discovery in Genome-wide Experimental Datasets. Nucleic Acids Res. 47 (D1), D607–D613. 10.1093/nar/gky1131 30476243 PMC6323986

[B28] Tang Z. Li C. Kang B. Gao G. Li C. Zhang Z. (2017). GEPIA: a Web Server for Cancer and normal Gene Expression Profiling and Interactive Analyses. Nucleic Acids Res. 45 (W1), W98–w102. 10.1093/nar/gkx247 28407145 PMC5570223

[B29] Tretina K. Park E.-S. Maminska A. MacMicking J. D. (2019). Interferon-induced Guanylate-Binding Proteins: Guardians of Host Defense in Health and Disease. J. Exp. Med. 216 (3), 482–500. 10.1084/jem.20182031 30755454 PMC6400534

[B30] Tripal P. Bauer M. Naschberger E. Mörtinger T. Hohenadl C. Cornali E. (2007). Unique Features of Different Members of the Human Guanylate-Binding Protein Family. J. Interferon Cytokine Res. 27 (1), 44–52. 10.1089/jir.2007.0086 17266443

[B31] Vestal D. J. Jeyaratnam J. A. (2011). The Guanylate-Binding Proteins: Emerging Insights into the Biochemical Properties and Functions of This Family of Large Interferon-Induced Guanosine Triphosphatase. J. Interferon Cytokine Res. 31 (1), 89–97. 10.1089/jir.2010.0102 21142871 PMC3021356

[B32] Vismara M. F. M. Donato A. Malara N. Presta I. Donato G. (2019). Immunotherapy in Gliomas: Are We Reckoning without the Innate Immunity. Int. J. Immunopathol Pharmacol. 33, 205873841984337. 10.1177/2058738419843378 PMC6458670 30968718

[B33] Wang X. Wang S.-s. Zhou L. Yu L. Zhang L.-m. (2016). A Network-Pathway Based Module Identification for Predicting the Prognosis of Ovarian Cancer Patients. J. Ovarian Res. 9 (1), 73. 10.1186/s13048-016-0285-0 27806724 PMC5093979

[B34] Warde-Farley D. Donaldson S. L. Comes O. Zuberi K. Badrawi R. Chao P. (2010). The GeneMANIA Prediction Server: Biological Network Integration for Gene Prioritization and Predicting Gene Function. Nucleic Acids Res. 38, W214–W220. 10.1093/nar/gkq537 20576703 PMC2896186

[B35] Yu C.-J. Chang K.-P. Chang Y.-J. Hsu C.-W. Liang Y. Yu J.-S. (2011). Identification of Guanylate-Binding Protein 1 as a Potential Oral Cancer Marker Involved in Cell Invasion Using Omics-Based Analysis. J. Proteome Res. 10 (8), 3778–3788. 10.1021/pr2004133 21714544

[B36] Yu S. Yu X. Sun L. Zheng Y. Chen L. Xu H. (2020). GBP2 Enhances Glioblastoma Invasion through Stat3/fibronectin Pathway. Oncogene 39 (27), 5042–5055. 10.1038/s41388-020-1348-7 32518375

[B37] Zhang J. Zhang Y. Wu W. Wang F. Liu X. Shui G. (2017). Guanylate-binding Protein 2 Regulates Drp1-Mediated Mitochondrial Fission to Suppress Breast Cancer Cell Invasion. Cell Death Dis. 8 (10), e3151. 10.1038/cddis.2017.559 29072687 PMC5680924

[B38] Zhang M. Wang X. Chen X. Zhang Q. Hong J. (2020). Novel Immune-Related Gene Signature for Risk Stratification and Prognosis of Survival in Lower-Grade Glioma. Front. Genet. 11, 363. 10.3389/fgene.2020.00363 32351547 PMC7174786

[B39] Zhang Y. Zhang Z. (2020). The History and Advances in Cancer Immunotherapy: Understanding the Characteristics of Tumor-Infiltrating Immune Cells and Their Therapeutic Implications. Cell Mol. Immunol. 17 (8), 807–821. 10.1038/s41423-020-0488-6 32612154 PMC7395159

[B40] Zhao J. Li X. Liu L. Cao J. Goscinski M. A. Fan H. (2019). Oncogenic Role of Guanylate Binding Protein 1 in Human Prostate Cancer. Front. Oncol. 9, 1494. 10.3389/fonc.2019.01494 31998647 PMC6967410

